# Auditory Sketches: Very Sparse Representations of Sounds Are Still Recognizable

**DOI:** 10.1371/journal.pone.0150313

**Published:** 2016-03-07

**Authors:** Vincent Isnard, Marine Taffou, Isabelle Viaud-Delmon, Clara Suied

**Affiliations:** 1 Espaces Acoustiques et Cognitifs, Sorbonne Universités, UPMC Univ Paris 06, CNRS, IRCAM, STMS, Paris, France; 2 Département Action et Cognition en Situation Opérationnelle, Institut de Recherche Biomédicale des Armées, Brétigny-sur-Orge, France; National University of Singapore, SINGAPORE

## Abstract

Sounds in our environment like voices, animal calls or musical instruments are easily recognized by human listeners. Understanding the key features underlying this robust sound recognition is an important question in auditory science. Here, we studied the recognition by human listeners of new classes of sounds: acoustic and auditory sketches, sounds that are severely impoverished but still recognizable. Starting from a time-frequency representation, a sketch is obtained by keeping only sparse elements of the original signal, here, by means of a simple peak-picking algorithm. Two time-frequency representations were compared: a biologically grounded one, the auditory spectrogram, which simulates peripheral auditory filtering, and a simple acoustic spectrogram, based on a Fourier transform. Three degrees of sparsity were also investigated. Listeners were asked to recognize the category to which a sketch sound belongs: singing voices, bird calls, musical instruments, and vehicle engine noises. Results showed that, with the exception of voice sounds, very sparse representations of sounds (10 features, or energy peaks, per second) could be recognized above chance. No clear differences could be observed between the acoustic and the auditory sketches. For the voice sounds, however, a completely different pattern of results emerged, with at-chance or even below-chance recognition performances, suggesting that the important features of the voice, whatever they are, were removed by the sketch process. Overall, these perceptual results were well correlated with a model of auditory distances, based on spectro-temporal excitation patterns (STEPs). This study confirms the potential of these new classes of sounds, acoustic and auditory sketches, to study sound recognition.

## Introduction

Although human listeners can apparently recognize very easily and with no effort very diverse sound sources in their surrounding environment, the literature focusing on the recognition of natural sounds and on the features used by the listeners to recognize them is relatively scant (e.g. [1,2]). Yet, as it has been argued for a long time in vision [3], natural stimuli may recruit specific mechanisms derived from adaptation to natural environments. The specificity of natural sounds has recently been highlighted: they can capture attention in an auditory-visual setting [4], and a few milliseconds are enough to recognize them [5–7]. The majority of studies focusing on the features used by the auditory system for the representation of natural sounds comes from brain imaging techniques. Until recently, a fairly accepted model of cortical processing of natural sounds implied a hierarchical temporal stream, from the encoding of low-level features to a high-level and more abstract category encoding [8,9]. It has been shown and developed for voice sounds [10], tool vs. animal sounds [11], or songbirds, animal sounds, speech and musical instruments [12]. Taking carefully into account some low-level acoustic features, other models have been proposed, involving distributed neural representations in the entire human auditory cortex for both low-level features and abstract category encoding [13–15]. They also showed that a complex spectro-temporal pattern of features represents more accurately the auditory encoding of natural sounds than a purely spectral or temporal approach (see [16] for animal sounds only; [13,17]; see also [18] for a computational and psychophysical approach). In particular, Moerel et al. [14] found that the voices and speech regions also responded to low-level features, with a bias toward low-frequencies that are characteristic of the human voices. This result is coherent with the theoretical approach proposed by Smith and Lewicki [19], which shows that the auditory code is optimum for natural sounds and especially suggests that the acoustic structure of speech could be adapted to the physiology of the peripheral auditory system.

As evidenced in this theoretical approach [19], or in physiological studies [20], not all information in a sound is useful to encode natural sounds: sparse coding based on the time/frequency properties of the auditory system is a highly efficient coding strategy. In perceptual studies, this is a well-known fact, not all information is useful for a given listening task. As primarily shown in speech studies, the auditory signal can be drastically distorted, or modified, and still be recognizable [21,22]. More recently, similar noise-band vocoder method as the one used by Shannon et al. [21], which removed most of the fine frequency information, has been applied to environmental sounds [2]. Although the effect is less spectacular with environmental sounds than with speech, the authors also showed that environmental sounds are resilient to a large amount of distortions. However, all of these transformations are not particularly sparse.

**Fig 1:**
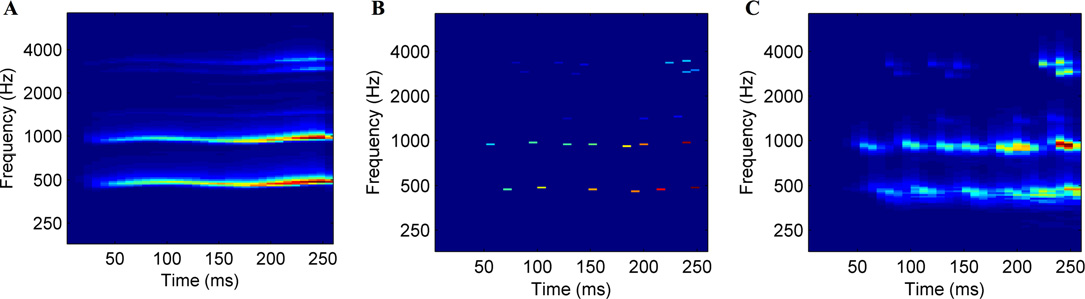
The sketch process. (A) The panel shows the first step, i.e. the time-frequency representation of a sound; here, the auditory spectrogram of the original sound (a voice sound of a female alto singer singing an /a/ on a B4). (B) The panel represents the sparsification algorithm: the 25 highest peaks in the signal are selected, corresponding to the 100 feat./s sparsification level. Based on this sparse representation, a sketch sound is then resynthesized. (C) The panel displays the auditory spectrogram of this sketch sound.

Recently, Suied et al. [23] have tackled the question of the sounds features that carry the most substantial information for a listener by introducing a new behavioral method: auditory sketches. Sketches are sparse representations of sounds that are severely impoverished, but still afford good performance on a given perceptual task, for example, recognition (see Fig 1 for an illustration of the sketch process). In order to create an auditory sketch, the first necessary step is to choose the appropriate representation of the sound. Because auditory representations are inspired by the physiology of the auditory system, they should contain the features relevant to perception. The second step consists in selecting sparse features on these representations. Again, if the auditory representation is efficient, the selection mechanism should be drastic. Finally, the representation was then inverted to give rise to a new sound: the auditory sketch. A proof of concept of the auditory sketch process was done in a first study [21], by studying the recognition of different emotions in voices. For this particular task, good recognition of the auditory sketches was observed. Nevertheless, the hypothesis that biologically plausible representations are better suited for efficient sketches than simple time-frequency representations remains to be tested. In addition, an extension of these results to more various sound sources is needed in order to generalize the sketch process. Finally, no attempt to model the acoustic features present in the sketches, which enable a good recognition, had been made.

The aim of the present study was to test the auditory sketches idea with a large, diverse, but still controlled set of sounds, and, by this mean, try to untangle the acoustic features used by the listeners to recognize these sounds. Listeners were presented with sketch sounds and had to recognize them, by indicating the category to which they belong. Four sound categories were used: voices, instruments, birds, and vehicles sounds. The original sounds of two of these four categories (voices and instruments) were equalized in pitch, loudness, and duration. At least for these two categories, listeners were thus left with only timbre cues to perform the task (see [24–26]). Adding the other two categories (birds and vehicles) ensured a sufficiently heterogeneous set of sounds in order to test the generality of the sketch process, but still controlled, by measuring some of the classical timbre cues highlighted in previous studies on timbre perception, like spectral centroid (see [27]), or in previous imaging studies on the encoding of natural sounds by the auditory cortex, like the Harmonic-to-Noise Ratio, or HNR (e.g. [13,15]). To test whether biologically grounded representations lead to more efficient sketches, we compared two time-frequency representations: an auditory spectrogram (see [28]), and a classical acoustic spectrogram, which performs a Fourier transform. The two resulting classes of sketches sounds will be referred to as ‘auditory sketches’ and ‘acoustic sketches’. The same sparsification levels as in the first study (10, 100, and 1000 features/second—the ‘features’ being here the energy peaks) were also tested, in order to explore how the recognition evolves (positively, we hypothesize) with the increase of the number of features.

## Experiment

### Methods

#### Participants

Fourteen individuals (6 men and 8 women; mean age 24.4 ± 2.7) took part in this experiment. None of the individuals reported having hearing problems. They all provided written informed consent to participate in the study. The Institutional Review Board of the French Institute of Medical Research and Health ethically approved this specific study prior to the experiment (opinion n°14–151). All participants were paid for their participation.

#### Original and sketch sounds

120 original sounds were used, equally divided into four categories: instruments, birds, vehicles, and voices (30 different sound exemplars in each category). These original sounds were selected in the Sound Ideas database (vehicle and bird sounds), in the Vienna Library database (instrument sounds), and in the RWC database (voice sounds). As in the Giordano et al.’s study [15], the sound set was characterized in terms of pitch, HNR and spectral centroid. Ten different instruments were selected: celesta, bassoon, flute, harp, clarinet, marimba, oboe, trumpet, cello, and vibraphone. Each instrument was played at 3 different pitches (F4, G#4, and B4), leading to 30 instrument sounds. For the voices, 5 different vowels were chosen (/a/, /e/, /i/, /o/, and /u/), each sung by a male tenor singer or a female alto singer. Vowels were sung at the same pitches as the instrument sounds (F4, G#4, and B4). Their Harmonic-to-Noise Ratio (HNR) was estimated using Praat software [29]. The HNR measures the ratio of the periodic and aperiodic (noisy) components of a signal. The mean (± standard deviation, SD) HNR value for the voices was 22.3 dB ± 9.1 dB; for the instruments, it was 26.6 dB ± 5.9 dB. A one-way ANOVA was run to compare the mean HNR of the four categories, and it revealed a significant effect of the HNR [F(3,116) = 87,35; p<0.0001; see below for the other two categories]. Tukey-HSD post-hoc tests showed that there was no statistical difference between the HNRs of the voices and the instruments [p = 0.07]. The 30 bird sounds were composed of a variety of birds: e.g. blue jay, crow, eagle, flycatcher. The pitch values for each of these 30 sounds were estimated using Praat, by means of an autocorrelation method. The pitch estimates ranged from 308.9 Hz to 582.8 Hz with a mean of 491.0 Hz (± 76.2 Hz). The average pitch for birds was slightly higher than the pitches of voices and instruments [t = 4.03; p<0.0002]. The mean HNR (± SD) for bird sounds was 11.5 dB (± 6.0 dB), lower than the HNR for voices and instruments, but higher than the HNR for vehicles (see below) [Tukey HSD post-hoc tests: p<0.0002 in all cases]. The vehicle sounds were running engine sounds. Because of their noisy nature, pitch could not be estimated for the vehicle sounds; with the exception of a few of them, for which the pitch obtained was apparently lower than the pitch of the other categories (around 180 Hz, compared to an average around 450 Hz for the three other categories). The HNR estimate of the vehicle sounds had a mean of 0.3 dB ± 6.2 dB. It was lower than for all the other categories [Tukey-HSD post-hoc tests: p<0.0002 in all cases]. Instruments and voices were comparable in terms of spectral centroid because of their similar harmonic structure (respectively: 955 Hz ± 495 Hz and 943 Hz ± 545 Hz), whereas it was much higher for birds [Tukey-HSD post-hoc tests: p<0.0002 in all cases], although with an important variance from one sound to the other (3122 Hz ± 1193 Hz). Spectral centroid of the vehicles was comparable to that of instruments and voices, with a mean of 719 Hz ± 449 Hz. Finally, all 120 sounds were equalized in duration (250 ms). The vehicle sounds were almost stationary and we arbitrarily chose 250-ms excerpts in the sounds (with 10-ms fades in and out to prevent clicks). For the bird, instrument, and voice sounds, the first 250 ms of the signal were kept, thus preserving their natural attack. 10-ms fade-outs were applied to these sounds. The sounds had a sampling frequency of 16 kHz, so there was no energy above 8 kHz.

These 120 original sounds were then transformed in acoustic and auditory sketches, following the method outlined in the Introduction: peaks were selected on an acoustic or auditory time-frequency representation of the sound, and then resynthesized in a new sound: the sketch (see Fig 1). Six sketch conditions were created: two representations (acoustic and auditory), and three levels of sparsity (10, 100, and 1000 features/second), leading to a total of 720 sketch sounds. A set of sound examples is available at: https://hal.archives-ouvertes.fr/hal-01250175.

**Fig 2:**
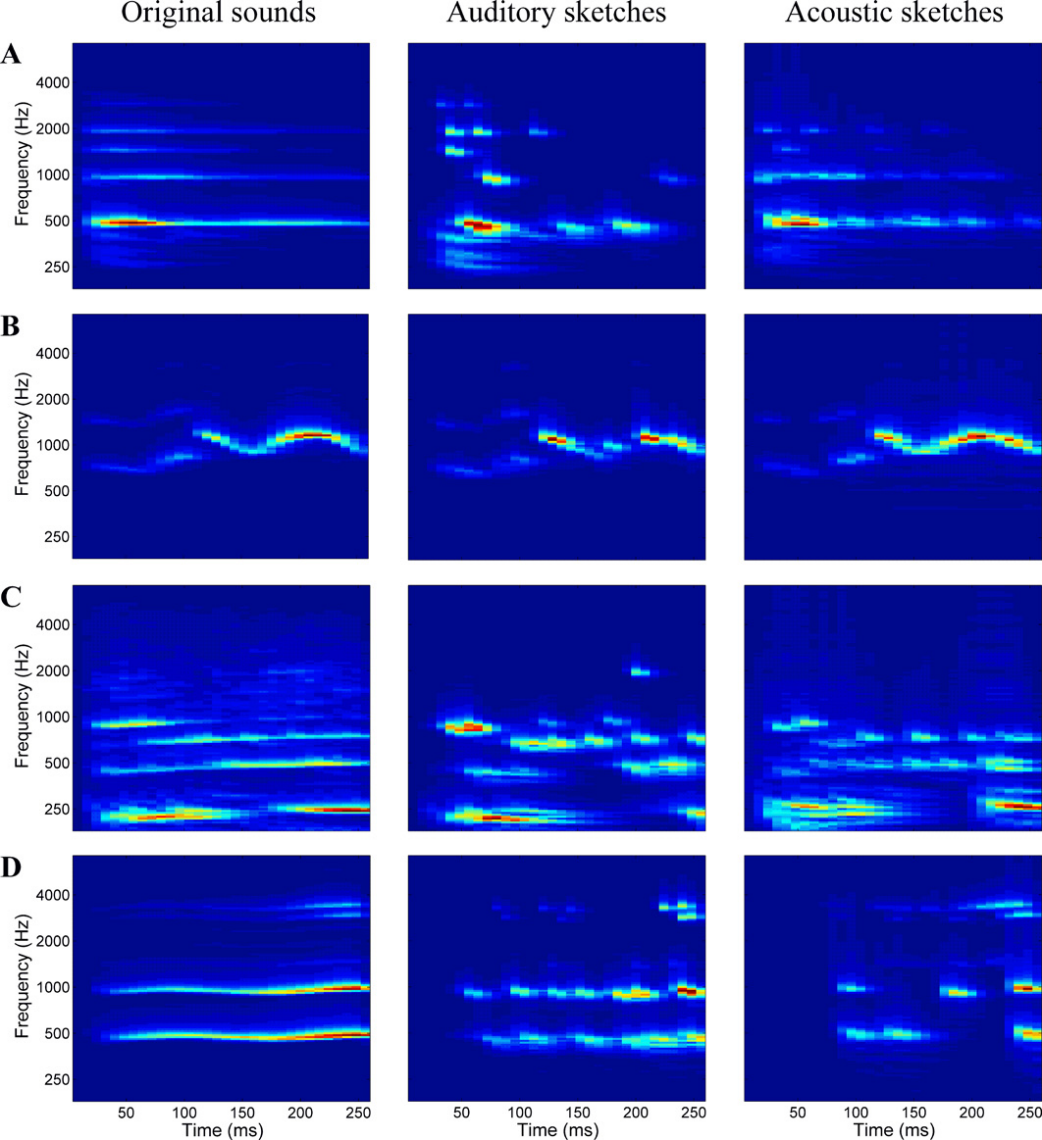
Auditory spectrograms of original and sketch stimuli. All panels are auditory time-frequency representations (Chi et al., 2005; see Original and sketch sounds section) of original and sketch stimuli. Left: original sounds; middle: auditory sketches (100 feat./s); right: acoustic sketches (100 feat./s). The sound examples are from the categories: (A) instruments: a harp playing a B4, (B) birds: a loon vocalization, (C) vehicles: a motorcycle, (D) voices: a female voice, singing the vowel /a/, B4.

The acoustic spectrogram was performed with fast Fourier transform on 8-ms Hanning windows. The auditory spectrogram mimics the frequency decomposition performed by the cochlea. It was obtained with 128 overlapping constant band-pass filters with center frequencies uniformly distributed along a logarithmic frequency axis, followed by spectral sharpening simulating lateral inhibition (1st order derivative and half-wave rectifier) [28]. The original programs are freely available online as the "NSL toolbox" (http://www.isr.umd.edu/Labs/NSL/Software.htm). Temporal integration was performed with 8-ms time windows. The resulting matrices for both representations had a similar size of 128 frequency bins x 32 temporal samples. The selection of features performed on these representations was based on the peak-picking of local maxima (see [23]). Here, the features were energy peaks. First, all local maxima were identified. Then, they were sorted by decreasing order, and only the n largest were kept; n corresponding to the sparsification level. This algorithm, by using a simple local maxima detection, tended to select relatively distant energy peaks, as high-energy areas in the original time-frequency representations could be summarized in one peak (for an illustration of the peak-picking effect, see also Fig 2). The same three levels of sparsification as in the previous study [23] were chosen: 10, 100 and 1000 features per second. For the 250-ms stimuli of the current experiment, it means that 3, 25 or 250 energy peaks were kept. However, for some of the sounds, the total number of peaks was smaller than 250 (more precisely, for 39% of the sounds, for which the mean number of peaks was 167±48). This was the case for all the auditory sketches of the instrument sounds (M = 155±39), 4 acoustic sketches of the instrument sounds (M = 213±30), 24 auditory sketches of the bird sounds (M = 205±29), 4 auditory sketches of the vehicle sounds (M = 232±9), all auditory sketches of the voice sounds (M = 130±36), and 2 acoustic sketches of the voice sounds (M = 227±3).

Then, the sparse representation was inverted back to give rise to the sketches. For the acoustic sketches, reconstruction was possible by simple inverse fast Fourier transform. The selected acoustic features were converted back to the amplitudes and phases stemming from the analyses of the original signals, to be resynthesized in acoustic sketches. For the auditory sketches, because of the nonlinear processing (lateral inhibition, thresholding), direct reconstruction could not be achieved. Similarly as in [23], we used the method developed by Yang et al. [30], which provides reconstruction for auditory spectrogram that are perceptually similar to the original, whenever there is no specific treatment on the auditory spectrogram. The algorithm estimates the phases thanks to an iterative procedure, which starts with a Gaussian distributed white noise and reconstructs the time waveform by inverse filtering.

Finally, the 840 (720 sketches and 120 original) stimuli were normalized by the root-mean-square level. Examples of original sounds and sketches, from each category, are represented in Fig 2.

#### Apparatus

The stimuli were presented diotically through a Sennheiser HD 250 Linear II headphone, connected to a RME Fireface 800 digital-to-analog converter, at a 16 kHz sampling rate. They were presented at around 68 dBA. The experimental session was run using a Matlab R2008b interface on an Apple Mac Pro. The participants were tested individually in a double-walled IAC sound-proof booth. They provided their response using the computer mouse, by clicking on the corresponding button on a computer screen.

#### Procedure

A four-alternative forced choice (4-AFC) paradigm was used. On each trial, participants heard a single sound, which could be either an instrument, a bird, a vehicle or a voice sound. They had to classify the sound they just heard into one of the four categories. No feedback was provided during the test sessions, only during the short training blocks.

For the main experiment, only sketch sounds were used. We carefully avoided familiarizing the participants with the original sounds at the beginning, to ensure that the first encounter with each sound was with its sketch version. For each of the six sketch conditions (2 time-frequency representations x 3 sparsification levels), and for each of the categories, 30 repetitions (each corresponding to a different sound; see Stimuli section) were collected. These 720 trials were presented in a randomized manner. Breaks were possible every 180 trials. Before data collection began, participants performed a short training block. The training block contained one example of a sketch sound for each category and for each condition, leading to 24 stimuli in total. Sounds for the training were not included in the main experiment stimulus dataset. At the end of the main experiment, a control block was run on the original stimuli alone, to ensure that the original sounds were well recognized. The order of presentation of the 120 original stimuli was randomized within a unique block of 120 stimuli. The four original sounds that were used to generate the sketches sounds of the first training session were also presented at the beginning of the second block, as a small training. The total experiment lasted about two hours.

#### Statistical analyses: signal detection model

To evaluate performance, the d' statistic of signal detection theory (SDT) was used. However, because the original theory has been developed for tasks with only 2 possible responses (yes/no, or 2-AFC; see [31]), we had to extend the theory to a 4-AFC case.

The traditional approach to apply SDT to m-AFC tasks (here, m = 4) assumes that there is no response bias [31]. However, it has been shown that this can affect the d' computations [32]. The method described by DeCarlo [32] takes into account the biases for each possible choice, in order to compute a global d' score for m-AFC tasks. It means that, although biases are computed for each choice (here, the four categories), only an average d' would be available. We thus extended DeCarlo’s method to compute d' scores for each category, and in each sparsification condition, while still taking the biases into account.

The decision rule here was the same as in DeCarlo [32], whereas the structural model, differing from DeCarlo [32], included the d' scores for each category as variables. We derived from the decision rule and the structural model a set of three equations which constitutes our 4-AFC model with bias in a normal theory version,

$$p(Y=1|X_{1},X_{2},X_{3})=\int_{-\infty }^{\infty }\Phi (d_{1}X_{1}-d_{2}X_{2}+b_{1}-b_{2}+e_{1})\Phi (d_{1}X_{1}-d_{3}X_{3}+b_{1}-b_{3}+e_{1})\Phi (d_{1}X_{1}-d_{4}X_{4}+b_{1}+e_{1})f(e_{1})d(e_{1}),$$

$$p(Y=2|X_{1},X_{2},X_{3})=\int_{-\infty }^{\infty }\Phi (d_{2}X_{2}-d_{1}X_{1}+b_{2}-b_{1}+e_{2})\Phi (d_{2}X_{2}-d_{3}X_{3}+b_{2}-b_{3}+e_{2})\Phi (d_{2}X_{2}-d_{4}X_{4}+b_{2}+e_{2})f(e_{2})d(e_{2}),$$

$$p(Y=3|X_{1},X_{2},X_{3})=\int_{-\infty }^{\infty }\Phi (d_{3}X_{3}-d_{1}X_{1}+b_{3}-b_{1}+e_{3})\Phi (d_{3}X_{3}-d_{2}X_{2}+b_{3}-b_{2}+e_{3})\Phi (d_{3}X_{3}-d_{4}X_{4}+b_{3}+e_{3})f(e_{3})d(e_{3}).$$

To limit the number of variables in our set of equations, the bias scores computed with DeCarlo’s method [32] were used as inputs in our version of the model. The four d' scores, corresponding to the four categories, were the variables. Both models were fitted thanks to OpenBUGS programs. They were run for each dataset with 4000 burnins and 16000 iterations. With this amount of iterations, the Monte Carlo errors were less than 5% of the posterior standard deviation, which is the criterion suggested for convergence (cf. [32]).

All the statistical tests (repeated-measures ANOVA) were conducted on these d' scores. Chance level corresponds to a d' of 0, while near perfect recognition (here, proportion correct of 97%) corresponds to a d' of 3.2.

### Results

#### Outlier sounds and participants

The sounds, in their original version, were overall well recognized by participants (97.2% ± 8.4%) except for three sounds (two bird sounds and one vehicle sound), which were misidentified by more than 30% of participants. The results for the original and simplified versions of these sounds were excluded from the analyses.

For each participant, a recognition score for the original stimuli was computed. The mean recognition score of the 14 participants with the original stimuli, excluding the three outlier sounds, was 98.3% (SD = 4.7%). We also computed recognition scores for each participant on the 702 remaining simplified stimuli (M = 51.4% ± 5.7%). One participant had a particularly low recognition score with the simplified stimuli (< mean– 2xSD) and was thus excluded from the following analyses. The mean recognition score on the 13 remaining participants for the simplified stimuli was 52.4% (SD = 4.3%).

#### Global recognition of the simplified stimuli

**Fig 3:**
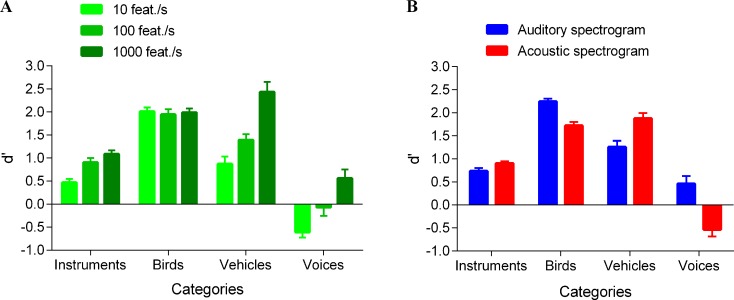
Recognition performance. (A) For each category, performance (as measured by d') is displayed at each sparsification level. With the exception of voice sounds, performance was well above chance even at the highest sparsification level, 10 feat./s. For voice sounds, performance was at chance or even lower (negative d'), meaning that participants responded systematically anything but voices for these voice sounds. (B) Performance is displayed for auditory sketches and acoustic sketches. For bird and voice stimuli, performances were higher with auditory sketches, whereas for vehicles, the reverse pattern emerged. No differences were observed for instrument sounds. Error bars correspond to the standard error of the mean.

A repeated-measures ANOVA with the sparsification level, the category, and the time-frequency representation as within-subjects variables was performed on the d' scores. Significant effects were further analyzed with Tukey-HSD post-hoc tests. Fig 3 displays the recognition performances for each category and each sparsification level.

It first revealed a significant main effect of the sparsification level [F(2,24) = 31.447; p<0.00001; η_p_^2^ = 0.724]. Participants better recognized sounds with low level (1000 feat./s) than medium level (100 feat./s) of sparsification [p<0.0006]. They also better recognized sounds with medium level (100 feat./s) than high level (10 feat./s) of sparsification [p<0.007].

The repeated-measures ANOVA also revealed a significant main effect of the time-frequency representation variable [F(1,12) = 7.188; p<0.03; η_p_^2^ = 0.375]. Recognition performances were better with auditory sketches than with acoustic sketches. However, this difference [p<0.03] was very small (Δd' = 0.2).

A significant main effect of the category was also found [F(3,36) = 108.810; p<0.00001; η_p_^2^ = 0.901]. Recognition performances were higher for the bird sounds than for the vehicle sounds [p<0.008]; higher for the vehicle sounds than for the instrument sounds [p<0.0002]; and higher for the instrument sounds than for the voice sounds [p<0.0002].

The ANOVA exhibited a significant two-way interaction between category and time-frequency representation variables [F(3,36) = 23.552; p<0.00001; η_p_^2^ = 0.663]. Participants better recognized the auditory sketch versions of bird and voice sounds than the acoustic sketch versions [respectively: p<0.03 and p<0.0002]. In contrast, they better recognized acoustic sketches of vehicle sounds [p<0.004]. For the instrument sounds, recognition performances were similar in both sketch conditions [p = 0.941].

Finally, the ANOVA revealed a significant two-way interaction between category and sparsification level [F(6,72) = 8.023; p<0.00001; η_p_^2^ = 0.401] (see Fig 3). For the vehicles, the recognition performances were better with a low level (1000 feat./s) than with medium level (100 feat./s) and high level (10 feat./s) of sparsification [p<0.0002 in both cases]. Similarly, for the voices, recognition performances were better with a low level (1000 feat./s) than with a medium level (100 feat./s) or high level (10 feat./s) of sparsification [respectively: p<0.04 and p<0.0002]. For the instruments, the recognition performances were better with low level (1000 feat./s) than with high level (10 feat./s) of sparsification [p<0.04]. For the birds, recognition performances were not influenced by the sparsification levels [p = 1.000 in all cases].

The effect of the two-way interaction between time-frequency representation and sparsification level variables was not significant [F(2,24) = 0.971; p = 0.393; η_p_^2^ = 0.075], nor was the three-way interaction [F(6,72) = 1.321; p = 0.259; η_p_^2^ = 0.099].

Finally, to investigate the sparsity levels for which recognition was above chance, we performed one-sample t-tests testing d' against 0 (chance level) for all conditions. Instrument and bird sketches were all significantly recognized above chance [one-sample t-tests: p<0.002 in all cases]. For vehicle sketches, they were also all recognized significantly above chance [p<0.00001 for all cases except the auditory sketches at 10 feat./s; p = 0.05]. Finally, for the voices, recognition performance was significantly above chance only for auditory sketches with a low sparsity level (1000 feat./s) [p<0.0003]. Unexpectedly, recognition was significantly below chance for acoustic sketches at 10 feat./s [p<0.00001]. This means that participants classified systematically the voice stimuli in any other category but not the voice one.

## Acoustic Analyses: Auditory Distance Model

To understand the possible acoustical bases of the perceptual results described above, we derived a new model of auditory similarity, based on the model developed by Agus et al. [33]. The original model computes auditory distances between two different sound categories. The model is based on the time-frequency distribution of energy for each sound, estimated using spectro-temporal excitation patterns (STEPs; [34]), which simulate peripheral auditory filtering. Auditory distances are then computed between pairs of STEPs, using a dynamic time-warping algorithm, to minimize the possible misalignment between features. Several behavioral results emerged from our data: we thus computed several auditory distances to investigate their auditory bases.

Firstly, in order to evaluate the impact of the sparsification level on the auditory distances, auditory distances between the original version of the sound and each of the sparsification level were computed. This was done for both acoustic and auditory sketches. The auditory distance between a sketch and its original version increased with the degree of sparsity: the higher the degree of sparsity, the larger the distance was [F(2,478) = 256.32; p<0.00001; η_p_^2^ = 0.51]. The mean distances were 0.21 ± 0.07 at 1000 feat./s; 0.29 ± 0.12 at 100 feat./s, and 0.37 ± 0.16 at 10 feat./s. This result mirrors the behavioral effect: the closer (in auditory distance terms) a sound was to its original version, the easier it was to recognize.

Secondly, we evaluated the effect of the time-frequency representation used as a basis for sparsification. We thus computed the distances between acoustic and auditory sketches, for each category. The auditory distance between an acoustic sketch and an auditory sketch depended on the category [F(3,267) = 30.061; p<0.00001; η_p_^2^ = 0.25], with slightly (as revealed by the small η_p_^2^) lower auditory distances for the birds than for the three other categories [Tukey-HSD post-hoc test: p<0.00001]. The mean distances were 0.20 ± 0.06 for instruments, 0.17 ± 0.07 for birds, 0.26 ± 0.05 for vehicles, 0.21 ± 0.07 for voices. Overall, no large differences between acoustical and auditory sketches emerged, as a function of the category. There was an acoustical difference between both types of sketches, but this auditory model distance could not explain the pattern of results observed in the perceptual results (see Fig 3B).

**Fig 4:**
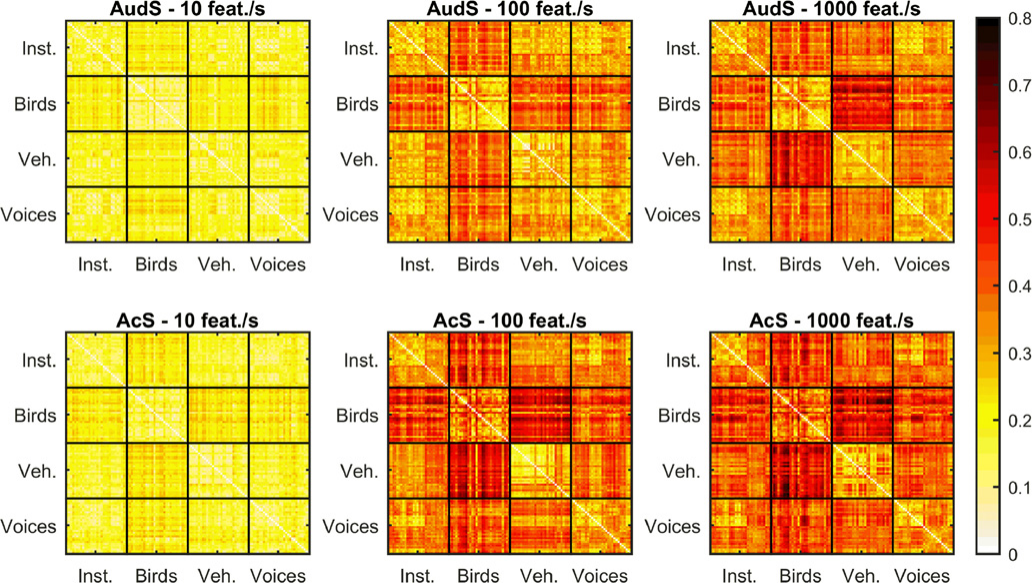
Auditory distance model. For each time-frequency representation (AudS: auditory spectrogram, and AcS: acoustic spectrogram) and each sparsification level (10, 100, and 1000 features per second), an auditory distance dissimilarity matrix is plotted (see [33]). The mean absolute distance between STEPs [34] is represented for each sound pair of each category (Inst. for musical instruments, Birds, Veh. for vehicle engine sounds, and Voices). With the high level of sparsity (10 feat./s), sounds are more similar between them than with the low level of sparsity (1000 feat./s). No obvious differences emerged between the two auditory or acoustic time-frequency representations.

Finally, in order to model the entire set of data, we computed distances between categories, for each sparsification level and each type of sketch. This model is probably the most accurate to model our data, because it allows us to compare the auditory distances between categories (as a function of sparsification level and time-frequency representation) with the d' scores, which are themselves ‘perceptual’ distances between the categories, for the 4-AFC task. Each category was considered successively as a target category (30 sounds), while the three remaining categories were considered as distractor categories (90 sounds). For each condition (2 time-frequency representations x 3 sparsification level), we computed the auditory distance Ad between each stimulus and stimuli from the other categories with the following equation: Ad(*i*) = μ_*distr*_(*i*) − μ_*targ*_(*i*),*i* = 1 … 30, where *i* is a stimulus of the target category, μ_*distr*_ is the mean of the distances between the target stimulus and each stimulus of the distractor categories (90 distances for each target stimulus), and μ_*targ*_ is the mean of the distances between the target stimulus and the other stimuli of the target category (29 distances for each stimulus). The six distance matrices are represented in Fig 4.

**Fig 5:**
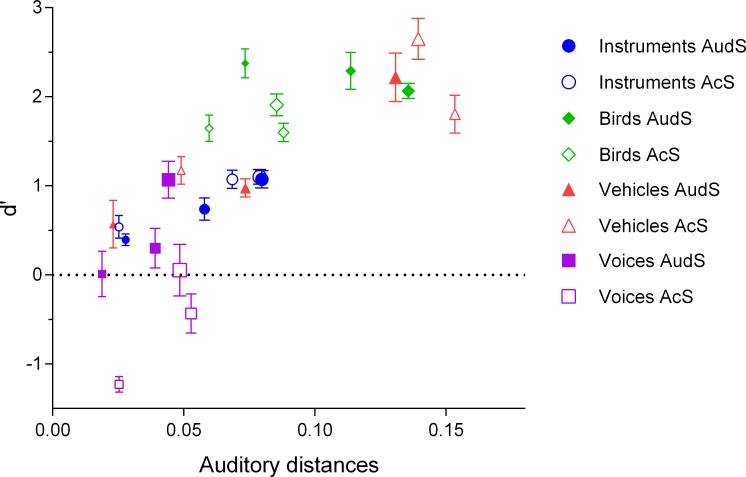
The perceptual results (d') plotted as a function of the auditory distance values. The filled symbols represent sketches based on the auditory spectrogram (AudS) representation; the open symbols are for the acoustic spectrogram (AcS) representation. The size of the symbols corresponds to the level of sparsity: small symbols for 10 feat/s; medium symbols for 100 feat/s; large symbols for 1000 feat/s. Error bars correspond to the standard error of the mean. A good correlation is exhibited between the model and the data.

To compare the auditory distance matrices with the perceptual results (d'), we computed, for each category, the mean of the auditory distances Ad on all the stimuli of the category. Fig 5 displays the d' scores for each condition as a function of the corresponding auditory distances for these conditions. The perceptual results were overall well correlated with the auditory distances: the more sounds were similar (smaller auditory distances), the more they were difficult to recognize (Spearman's correlation: ρ = 0.721, p<0.00001).

## Discussion

We have studied acoustic and auditory sketches, new classes of sounds based on sparse representations, which are severely impoverished versions of original sounds. Salient features of the sounds were kept by means of a simple peak-picking algorithm that was performed on a time-frequency representation. Two representations were compared: an auditory spectrogram, i.e. a biologically grounded representation, and an acoustic spectrogram. Three levels of ‘sparsification’, i.e. the number of energy peaks kept in the representation, were also contrasted. To test the sketch process, we conducted an experiment that assessed the recognition by human listeners of acoustic and auditory sketches. We found that: (1) with the notable exception of voice sounds (see (3)), all sounds were recognized above chance even when they were drastically impoverished, i.e. with a very low number of features (10 feat./s). In addition, recognition performance increased with the increase of the number of features; (2) in contrast to our hypothesis, no clear differences in recognition were observed between the acoustic and the auditory sketches; (3) voice sounds followed a very different response pattern than all the other categories, with performance being at chance, or even below chance, meaning that participants systematically recognized voice sounds as any other categories except voice itself; (4) a model based on auditory distances between spectro-temporal excitation patterns (STEPs) exhibited a good correlation with the perceptual data.

In our experiment, for all sounds except voice sounds, extreme sparsification could be applied (only 3 peaks for one sound were kept in the most drastic conditions) while keeping recognition above chance. As has been shown previously for speech [21,22] and environmental [2] sounds, auditory recognition can be very robust to sound distortions and modifications. It is also worth noting that for the bird sound category, recognition plateaued already at 10 feat./s (with a relatively high d’, around 2). The few key features, probably located in the upper part of the spectrum (higher spectral centroid), were selected by the peak-picking algorithm even at the highest level of sparsification (10 feat./s). Within this set of sounds where the birds stand out with respect to these high-frequency features, adding more peaks did not add any necessary information for the listener. For all the other categories, as expected, as the number of features increased, so did recognition performances. Altogether, these results confirm that (very) sparse representations of sounds can produce perceptually relevant results.

However, the results of our experiment did not support the hypothesis that, for any type of natural sound, sparse representations would lead to better results if they are implemented on a biologically grounded representation of sounds, like an auditory spectrogram. For some categories (voices and birds), the behavioral advantage, evidenced by better performance, was indeed observed for the auditory sketches. However, no differences were found for the instrument sounds; and the reverse pattern—with higher performance for the acoustic sketches—appeared for the vehicle sounds. No simple explanation for this surprising interaction can be given based on the basic timbre features computed on the sounds (see Methods for the differences between the categories in the spectral centroid and the HNR). One of the limitations of our experiment is the short duration of the sound used (250 ms). With this duration, a possible difference between the two representations would not arise. The probability to have more potential features useful for recognition (and thus more important differences between the features kept in the two representations) is indeed higher with longer sounds, at least for sounds that are not stationary. It is interesting to note that the only category, for which performance was actually worse with the auditory sketches than with the acoustic sketches, is the only one that contained stationary sounds (vehicles). This is in accordance with a result obtained in the audio signal processing community (see [35]). They found that, whereas for typical steady-state signals the Fourier representation is sufficient to provide a good representation, for sounds with onsets and transients, like voices, animal calls, or music, a ‘union of bases’ composed of both a Modified Discrete Cosine Transform basis and a Wavelet basis, is needed to have a better sparse representation of the signal. Another way to interpret this result is in terms of a dichotomy between living and non-living sounds. Living sound (voices and birds) were better recognized when presented as auditory sketches, whereas non-living sounds did not show any advantage (or even show a disadvantage) when presented as auditory sketches. This interpretation remains to be confirmed and extended in future experiments.

The results obtained for the voice sounds, i.e. recognition at chance level or even negative d', can be seen as another behavioral evidence that voice is special (see [36] for the evidence for speech; [7,33] for behavioral evidences for voices; [37] for a review on the selectivity for the human voice observed in fMRI studies). The cues useful for voice recognition were completely removed when subjected to the sketch process. In Agus' study [33], using chimeric sounds in which the temporal structure from one sound (e.g. instrument) is mixed with the spectral structure of another (e.g. voice), they showed that only natural voices could elicit special behavioral advantage for voices; in their case, this advantage was provided by faster reaction times. In the present study, we showed that a large and diverse set of sounds could be simplified with only a few peaks, while still being recognized well above chance. The noticeable exception of voice sounds may suggest that for recognition of voices to be effective, complex spectro-temporal patterns might be needed.

Finally, whatever the features used to recognize sounds sparsified on different representations and with different levels of sparsification, the perceptual results were relatively well correlated with a model based on auditory distances of STEPs (see [33]). The larger the distances between one category and the other three, the better the recognition was. This new auditory distance model would probably be useful in future studies, using various techniques such as psychophysical methods and/or brain imagery, in a further attempt to equalize different classes of stimuli along some relevant acoustic or auditory dimensions (see for example [13,15]).

## Supporting Information

S1 Dataset D-primes for all participants. (XLSX)

S2 Dataset Auditory distances between each sound for each sparsification condition. (XLSX)
